# Gene Expression in Uterine Leiomyoma from Tumors Likely to Be Growing (from Black Women over 35) and Tumors Likely to Be Non-Growing (from White Women over 35)

**DOI:** 10.1371/journal.pone.0063909

**Published:** 2013-06-13

**Authors:** Barbara J. Davis, John I. Risinger, Gadisetti V. R. Chandramouli, Pierre R. Bushel, Donna Day Baird, Shyamal D. Peddada

**Affiliations:** 1 Biomedical Sciences, Cummings School of Veterinary Medicine at Tufts University, North Grafton, Massachusetts, United States of America; 2 Obstetrics Gynecology and Reproductive Biology, College of Human Medicine, Michigan State University, Grand Rapids, Michigan, United States of America; 3 Biostatistics Branch, National Institute of Environmental Health Sciences, NIH, Research Triangle Park, North Carolina, United States of America; 4 Epidemiology Branch, National Institute of Environmental Health Sciences, NIH, Research Triangle Park, North Carolina, United States of America; The University of Hong Kong, Queen Mary Hospital, Hong Kong

## Abstract

The study of uterine leiomyomata (fibroids) provides a unique opportunity to investigate the physiological and molecular determinants of hormone dependent tumor growth and spontaneous tumor regression. We conducted a longitudinal clinical study of premenopausal women with leiomyoma that showed significantly different growth rates between white and black women depending on their age. Growth rates for leiomyoma were on average much higher from older black women than for older white women, and we now report gene expression pattern differences in tumors from these two groups of study participants. Total RNA from 52 leiomyoma and 8 myometrial samples were analyzed using Affymetrix Gene Chip expression arrays. Gene expression data was first compared between all leiomyoma and normal myometrium and then between leiomyoma from older black women (age 35 or older) and from older white women. Genes that were found significant in pairwise comparisons were further analyzed for canonical pathways, networks and biological functions using the Ingenuity Pathway Analysis (IPA) software. Whereas our comparison of leiomyoma to myometrium produced a very large list of genes highly similar to numerous previous studies, distinct sets of genes and signaling pathways were identified in comparisons of older black and white women whose tumors were likely to be growing and non-growing, respectively. Key among these were genes associated with regulation of apoptosis. To our knowledge, this is the first study to compare two groups of tumors that are likely to have different growth rates in order to reveal molecular signals likely to be influential in tumor growth.

## Introduction

Uterine leiomyoma is a common, benign, monoclonal, diploid smooth muscle tumor with a frequent mutation in mediator complex subunit 12 (*MED12*) [1]. The development of this tumor is dependent on the presence of ovarian hormones, and women typically develop multiple leiomyomata over their reproductive lifespan. Leiomyomata become subclinical and regress after menopause. Based on these characteristics, we expected that leiomyomata would demonstrate homogeneous patterns of growth relative to the menstrual cycle and would provide a useful model by which to understand mechanisms of hormone-dependent tumor growth. Instead, in a longitudinal MRI-based study of leiomyoma growth (The Fibroid Growth Study) we found that leiomyomata are heterogeneous in nature and exhibit autonomous patterns of growth]. For example, leiomyoma growth rates ranged from −89% to +138% volume change over a 6 month interval and individual growth rates were variable among different tumors within the same woman. Growth rates of individual leiomyomata were also independent of tumor size, intrauterine location or the hormonal environment of the woman [2]. Moreover, their short-term patterns of growth revealed that many tumors exhibited growth spurts, followed by a period of no growth or spontaneous regression. Concurrent growth and regression in different tumors from the same woman could be seen [3].

This heterogeneous and variable behavior of tumors has significant consequences for translating physical information gained from the in-life study to a tissue-based molecular analysis of tumor growth. That is, despite having knowledge of a tumor's immediately previous pattern of growth, it is not possible to predict whether a tumor at a single point in time is in a state of growth, static, or regressing. Even with in-life measurements, the most that could be known about a single sample is that the tumor was previously growing, previously not growing or previously regressing. Histological biomarkers such as mitotic or apoptotic index may be considered useful ancillary aids to infer a tumor's state of growth, but there is no evidence that these histological markers correlate to the physical growth status of a tumor. Indeed, leiomyomata have notoriously low mitotic indices despite achieving great size. However, we did find in the clinical study that tumor growth rates were significantly different between Caucasian and black women depending on their age [2]. Younger white women and all black women, regardless of age, had similar leiomyoma growth rates; in contrast, growth rates of leiomyoma was significantly decreased in older white women. The age-dependent decline in growth rates in older white women could not be explained by differences in tumor size, location, menstrual cycle patterns, parity, or BMI.

Based on these data, we reasoned that comparing leiomyoma from women of different age/ethnicity groups would provide a means of indirectly comparing leiomyoma likely to have different growth rates. Thus, we hypothesized that comparing gene expression of uterine leiomyomata categorized by age and ethnicity of the host would serve to amplify those growth-related genes over the landscape of genes that differentiate tumors from normal tissue. Indeed, this analysis has revealed previously unidentified networks that are plausibly related to the growth and regression of this benign tumor with implications for therapeutic strategies.

## Results

A total of 52 leiomyomata from 12 women of different ages and 8 myometrial samples were analyzed using Affymetrix microarrays (Table 1). Using the gene expression data, comparisons were then made between all leiomyomata compared to all myometrium and then between tumors categorized by age/ethnicity as likely to be growing and non-growing.

**Table 1:** Total samples collected from the FGS study for microarray analysis.

	Number of Participants	Number of Tumors	Number of Myometrium Tissues
**Race**			
Black	8	40	4
White	3	4	0
Other	1	8	4
**Total**	12	52	8
**Age**			
Young (less than 35)	5	22	
Old (35 or older)	7	30	
**Age × Race**			
Black and young	4	18	
Black and old	4	22	
White and young	0	0	
White and old	3	8	
Other and young	1	4	

Other =  Unknown (Neither white nor black).

### Leiomyoma vs. Myometrium

A total of 9516 probe sets were found to be differentially expressed, with fold changes ranging from −14.60 to 73.93 with 1044 probe sets having a fold change greater than 2. The PCA plots using these differentially expressed genes (see Figure S1) suggest a clear separation of leiomyoma and normal myometrium samples. Table S1 lists significantly up-regulated genes and Table S2 lists all significantly down-regulated genes in the comparison of leiomyoma and myometrial samples which had an absolute fold change larger than 5. For a complete list of all 9516 significantly differentially expressed probe sets one may refer to Table S3, an EXCEL spreadsheet. Noteworthy, ionotropic glutamate receptor *AMPA2* (*GRIA2*) was the most highly up-regulated gene in leiomyoma compared to myometrium, consistent with studies conducted by Tsibris [4]. We also found significant differential expression of dermatopontin (*DPT*) and various isoforms of the fibrillar (I, III, V) and nonfibrillar collagen genes (IV and VI) in leiomoyoma compared to myometrium, findings that are also consistent across other microarray studies [5]. Genes significantly down-regulated in leiomyoma compared to myometrium included activating transcription factor 3 (*ATF3*), alcohol dehydrogenase 1b, EGF containing fibulin-like extracellular matrix protein 1, *FOS*, aldehyde dehydrogenase 1a1and prostaglandin endoperoxide synthase 2 (prostaglandin G/H synthase and cyclooxygenase 2 (COX-2)). We also saw significantly lower expression of *MED12* in tumor compared to myometrium, though the fold change was small. This is consistent with disruption of this gene by a somatic mutation commonly found in leiomyoma tumors [1].

To verify expression, the same samples were analyzed for gene expression using RT-PCR, including *COX-2*. Genes selected correlated with and supported the direction and relative significance of genes increased or decreased in the microarray analysis, indicating our methodology was robust (Table S4). The only exceptions were collagen 4a3 (*COL4A3*) and estrogen receptor 2 (*ESR2*), which had two probes on the microarray chip with opposite directions of expression. We also found the down-regulation of genes involved in prostaglandin synthesis, a finding also reported by Arslan, et al., 2005 [6] and that supports our original hypothesis that a fundamental pathology of uterine leiomyoma cells is aberrant prostaglandin signaling leading to a failed contractile smooth muscle cell phenotype [7].

Based on the genes that were differentially expressed between leiomyoma and myometrium, the top biological functions identified by IPA were related to cancer, specifically leiomyoma and uterine cancer, and the top canonical pathways identified were classified as molecular mechanisms of cancer. The top 15 canonical pathways identified by IPA included previously identified and well-known molecules associated with leiomyoma including *IGF1* and prolactin and also included previously undisclosed signaling pathways including axonal signaling, stathmin1, glioma signaling and Hepatocyte Growth Factor (Table 2). Differentially expressed genes most often found in these pathways included RAS-related small GTPases *RAF-1* and *RAC-2* and their downstream effector molecules.

**Table 2:** Top fifteen statistically significant canonical pathways identified in Leiomyoma compared to myometrium using the Ingenuity Pathway Analysis.

Ingenuity Canonical Pathways	−log (p-value)
Molecular Mechanisms of Cancer	1.03E01
Axonal Guidance Signaling	8.45E00
Breast Cancer Regulation by Stathmin1	6.76E00
Glioma Signaling	6.61E00
Prolactin Signaling	6.54E00
Renin-Angiotensin Signaling	5.83E00
fMLP Signaling in Neutrophils	5.78E00
Signaling by Rho Family GTPases	5.71E00
Endothelin-1 Signaling	5.66E00
HGF Signaling	5.6E00
Rac Signaling	5.28E00
EIF2 Signaling	5.09E00
IL-8 Signaling	4.97E00
Erythropoietin Signaling	4.94E00
IGF-1 Signaling	4.92E00

**Figure 1:**
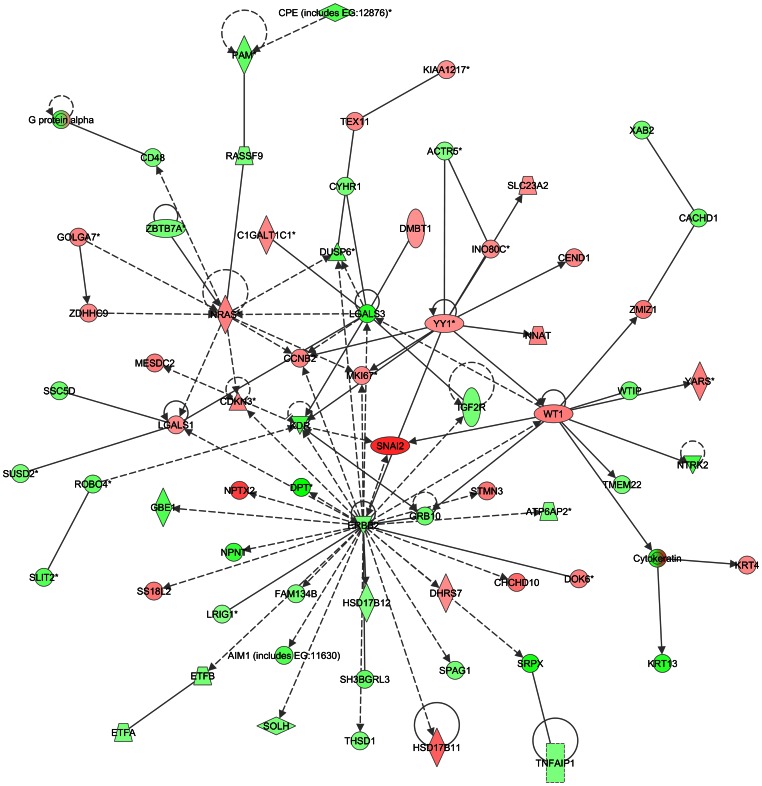
Network analysis of leiomyoma compared to myometrium – ERBB2 hub. Symbols in the figure represent the following: **Rectangle:** Nuclear receptor. **Oval:** Transcription regulator **Triangle:** Phosphatas. **Horizontal diamond:** Peptidase Vertical diamond: Enzyme. **Trapezium:** Transporter **Square with solid boundary:** Cytokine. **Square with dashed boundary:** Growth factor **Circle:** Other. **Solid line:** Direct interaction **Dashed line:** Indirect interaction. **Arrow:** Acts on **Horizontal “T”:** Inhibits.

According to the IPA analysis, one of the top 5 significant network signaling pathways in leiomyoma compared to myometrium was centered on down-regulation of *ERBB2* (Figure 1). Most of the genes closely linked to the *ERBB2* hub were also down-regulated, including dermatopontin (*DPT*). The pathway analysis identified *SNAIL2*, a mediator of epithelial-mesenchymal transition [8] as highly up-regulated, although this gene was not identified in our microarray data, a finding we attribute to the mesenchymal origins of smooth muscle and leiomyoma. In this network, decreased gene expression of *ERBB2* was also linked to up-regulation of *CCNB2*, the gene encoding cyclin B2, which was also up-regulated in our gene expression analysis. In animal models, knock-out of erbb receptors result in malformed hearts, and inhibition of erbb2 in adult mice results in myocardial dysfunction and inability of the cardiomyocyte to contract [9],[10],[11]. In human myocardiocytes in culture, maintenance of ERBB2 is critical for myofibrillar structure and function and therapies used for targeting ERBB2 positive breast cancers are associated with cardiotoxicity [10]. Thus, absence of *ERBB2* expression results in the inability of myocardiocytes to contract or appropriately differentiate. A similar mechanism may be at play in smooth muscle cells of leiomyoma where the decreased or absent gene expression of *ERBB2* underlies a defective contractile and differentiation signal. Particularly intriguing is the question as to what controls the expression of this gene. In leiomyoma, IPA shows a network connection between *ERBB2* and *DPT*, a relationship previously undisclosed in studies of uterine leiomyoma or, to our knowledge, in studies of breast cancer.

In summary, these results of the microarray analysis from leiomyoma compared to myometrial samples collected from the FGS show robust and consistent results with previously published studies and verified by RT-PCR. Given the validity of the sample set, we next sought to expand these analyses by comparing gene expression in tumors classified as growing and non-growing.

### Comparison of Leiomyomata from Older Black and Older White Premenopausal Women

For the tissues available for this microarray study, not all age groups were equally represented or reflective of the age groups in the initial analysis done in the FGS [2],[12]. Thus, for the tissues available for the microarray study, growth differences were re-evaluated based on dichotomizing age at 35 years old. The distinguishing growth rates in these age groups proved comparable to the initial study, in that there was a highly elevated odds of rapid growth (>20% increase in 3 months) for leiomyoma in black women older than 35 years compared to leiomyoma in white women older than 35 years. Among women of age 35 and older, the odds-ratio associated with rapid growth for a randomly chosen tumor from a black woman compared to a white woman was 2.8 with a 95% confidence interval (1.28, 6.00) and *p* = 0.01. Thus, tumors from black women aged 35 and older (“older black women”) were compared to tumors from white women of age 35 and older (“older white women”) to identify molecular factors that might reflect differential growth.

The individual genes in this comparison constituted distinct sets of up-regulated or down-regulated genes that generally differed from those that were differentially expressed between leiomyoma to myometrium (Tables 3 and 4). The PCA plots and heat map using these differentially expressed genes (see Figures S2 and S3) suggest a separation of leiomyoma samples from older black women compared to older white women. There was overlap in the identification of one gene in particular, *DPT*, which was significantly down-regulated in both the comparison between the two leiomyoma groups and in the comparison of all tumors to myometrium. Other genes found to be down-regulated in tumors from older black women compared to tumors from older white women included other matrix-related genes such as fibulin 1, calcium related migratory and endocytotic molecules netrin 1 and stoning 1, and genes involved in metabolism such as pyruvate dehyrogenase kinase isoenzyme IV. Genes significantly up-regulated only in the comparison of the two leiomyoma groups included muscle specific carbonic anhydrase III, which is important in buffering acid-base balance [13], and retinal pigment epithelium-specific protein, which is important in epithelial-mesenchymal transition in the fibrotic response [14].

**Table 3:** Top 25 significantly up-regulated genes in uterine leiomyoma from older black women compared to older white women.

Symbol	Entrez Gene Name	Location	Type(s)	p-value	Fold Change
CA3	carbonic anhydrase III, muscle specific	Cytoplasm	enzyme	6.0E-05	8.81
PSPH	phosphoserine phosphatase	Cytoplasm	phosphatase	1.2E-04	5.97
DAPP1	dual adaptor of phosphotyrosine and 3-phosphoinositides	Cytoplasm	other	1.20E-04	4.52
ANO1	anoctamin 1, calcium activated chloride channel	Plasma Membrane	ion channel	6.0E-05	4.19
CHRDL2	chordin-like 2	Extracellular Space	other	6.0E-05	3.98
C3orf70	chromosome 3 open reading frame 70	unknown	other	6.0E-05	3.92
COPG2IT1	COPG2 imprinted transcript 1 (non-protein coding)	unknown	other	6.0E-05	3.85
NCAM1	neural cell adhesion molecule 1	Plasma Membrane	other	2.5E-04	3.57
DAPP1	dual adaptor of phosphotyrosine and 3-phosphoinositides	Cytoplasm	other	1.9E-04	3.57
NME7	non-metastatic cells 7, protein expressed in (nucleoside-diphosphate kinase)	Cytoplasm	kinase	6.0E-05	3.36
PI15	peptidase inhibitor 15	Extracellular Space	other	3.6E-04	3.33
C3orf70	chromosome 3 open reading frame 70	unknown	other	6.0E-05	3.32
CRYBB2	crystallin, beta B2	unknown	other	6.0E-05	3.30
COL12A1	collagen, type XII, alpha 1	Extracellular Space	other	1.2E-04	3.29
UNC5D	unc-5 homolog D (C. elegans)	Plasma Membrane	other	6.9E-04	3.23
TUBE1	tubulin, epsilon 1	Cytoplasm	other	2.5E-04	3.10
NCAM1	neural cell adhesion molecule 1	Plasma Membrane	other	1.9E-04	3.08
LOC144481	hypothetical LOC144481	unknown	other	6.7E-04	2.92
SCN2A	sodium channel, voltage-gated, type II, alpha subunit	Plasma Membrane	ion channel	7.2E-04	2.90
SALL1	sal-like 1 (Drosophila)	Nucleus	transcription regulator	6.0E-05	2.89
FHOD3	formin homology 2 domain containing 3	unknown	other	6.0E-05	2.81
RIMS2	regulating synaptic membrane exocytosis 2	unknown	other	1.2E-04	2.80
COL12A1	collagen, type XII, alpha 1	Extracellular Space	other	2.5E-04	2.67
IGSF3	immunoglobulin superfamily, member 3	Plasma Membrane	other	5.8E-04	2.67

**Table 4:** Top 25 significantly down-regulated genes in uterine leiomyoma from older black women compared to older white women.

Symbol	Entrez Gene Name	Location	Type(s)	p-value	Fold Change
DPT	dermatopontin	Extracellular Space	other	1.2E-04	−5.45
TNFRSF21	tumor necrosis factor receptor superfamily, member 21	Plasma Membrane	other	6.0E-05	−4.52
CLEC2B	C-type lectin domain family 2, member B	Plasma Membrane	other	6.0E-05	−3.99
MX1	myxovirus (influenza virus) resistance 1, interferon-inducible protein p78 (mouse)	Nucleus	enzyme	6.0E-05	−3.83
TNFRSF21	tumor necrosis factor receptor superfamily, member 21	Plasma Membrane	other	6.0E-05	−3.75
DPT	dermatopontin	Extracellular Space	other	1.2E-04	−3.65
NDRG4	NDRG family member 4	unknown	other	5.9E-04	−3.64
MAP3K8	mitogen-activated protein kinase kinase kinase 8	Cytoplasm	kinase	1.2E-04	−3.61
STON1-GTF2A1L	STON1-GTF2A1L readthrough	Nucleus	transcription regulator	5.9E-04	−3.44
FBLN1	fibulin 1	Extracellular Space	other	1.2E-04	−3.40
DPT	dermatopontin	Extracellular Space	other	1.6E-04	−3.37
FBLN1	fibulin 1	Extracellular Space	other	2.7E-04	−3.28
PDK4	pyruvate dehydrogenase kinase, isozyme 4	Cytoplasm	kinase	2.2E-04	−3.19
IFIT3	interferon-induced protein with tetratricopeptide repeats 3	Cytoplasm	other	1.7–04	−3.13
GPRC5B	G protein-coupled receptor, family C, group 5, member B	Plasma Membrane	G-protein coupled receptor	6.0E-05	−3.06
GPR85	G protein-coupled receptor 85	Plasma Membrane	G-protein coupled receptor	6.0E-05	−3.04
CEBPD	CCAAT/enhancer binding protein (C/EBP), delta	Nucleus	transcription regulator	5.3E-04	−2.93
DDIT4	DNA-damage-inducible transcript 4	Cytoplasm	other	6.0E-05	−2.93
FLJ35700	hypothetical protein FLJ35700	unknown	other	6.0E-05	−2.93
GPRC5B	G protein-coupled receptor, family C, group 5, member B	Plasma Membrane	G-protein coupled receptor	6.0E-05	−2.88
EZR	ezrin	Plasma Membrane	other	2.5E-04	−2.81
RAP2C	RAP2C, member of RAS oncogene family	Cytoplasm	enzyme	7.2E-04	−2.79
FBLN1	fibulin 1	Extracellular Space	other	2.2E-04	−2.79
RAP2C	RAP2C, member of RAS oncogene family	Cytoplasm	enzyme	7.7E-04	−2.78
IFIT1	interferon-induced protein with tetratricopeptide repeats 1	Cytoplasm	other	8.0E-04	−2.77

Among the significant canonical pathways identified by IPA in leiomyoma from older black women (more likely to be growing) compared to leiomyoma from older white women (more likely to be non-growing) were interferon signaling and retinoic acid mediated apoptosis signaling, granzyme B signaling, and *RIG-1* pathways associated with a variety of early immune-mediated responses as well as stem cell and cancer cell survival responses (Table 5). The identification of these unique biological canonical pathways in the leiomyoma to leiomyoma comparisons is supportive of the physical findings that leiomyoma of older black women have generally different growth patterns than leiomyoma in older white women. These findings are also consistent with the idea that leiomyoma growth is promoted by an overall inhibition of apoptosis pathways.

**Table 5:** Statistically significant canonical pathways identified in IPA comparisons from older black women compared to older white women.

Ingenuity Canonical Pathways	−log(p-value)
Interferon Signaling	4.25E00
Retinoic acid Mediated Apoptosis Signaling	4.05E00
Hypoxia Signaling in the Cardiovascular System	3.1E00
Mitochondrial Dysfunction	2.58E00
DNA Double-Strand Break Repair by Non-Homologous End Joining	2.04E00
Purine Metabolism	2.01E00
Activation of IRF by Cytosolic Pattern Recognition Receptors	1.91E00
Granzyme B Signaling	1.87E00
Protein Ubiquitination Pathway	1.5E00
Death Receptor Signaling	1.42E00
Valine, Leucine and Isoleucine Biosynthesis	1.41E00
Role of RIG1-like Receptors in Antiviral Innate Immunity	1.38E00
Methionine Metabolism	1.31E00

**Figure 2:**
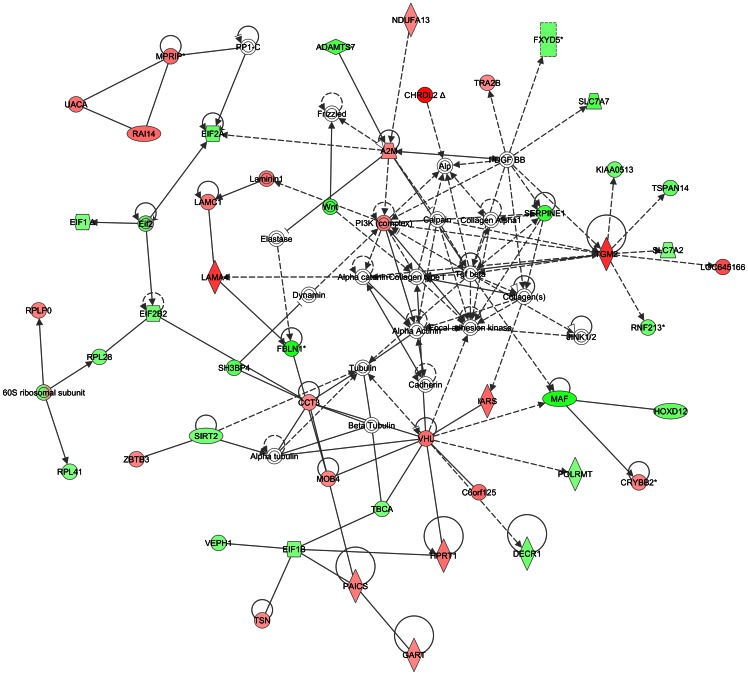
Network analysis of growing tumors compared to non-growing tumors – EGFR hub. Symbols in the figure represent the following: **Rectangle:** Nuclear receptor. **Oval:** Transcription regulator **Triangle:** Phosphatase. **Horizontal diamond:** Peptidase **Vertical diamond:** Enzyme. **Trapezium:** Transporter **Square with solid boundary:** Cytokine. **Square with dashed boundary:** Growth factor **Circle:** Other. **Solid line:** Direct interaction **Dashed line:** Indirect interaction. **Arrow:** Acts on **Horizontal “T”:** Inhibits.

Expanding on the relationships between these pathways, IPA identified *VHL*, a master regulator of a cells response to hypoxia, among the top five significant networks in leiomyomata from older black women (“growing” tumors) (Figure 2). Because most of the research involving *VHL* focuses on its loss of function and consequent cancer development, finding *VHL* up-regulated in growing leiomyoma appeared counterintuitive. However, significant up-regulation of *VHL* occurs in renal carcinoma cells and cytotrophoblasts under hypoxic conditions [15] and in renal proximal tubule epithelial cells under ischemia/reperfusion injury [16]. VHL protein (pVHL) also plays an important role in microtubule stabilization and growth promoting rescue events [17],[18], reflected in the network associations in leiomyoma from older black women. *VHL* is also networked with many collagens, which may reflect another function of pVHL in promoting cell interaction with extracellular matrix [19]. This later function has recently been shown as a fundamental mechanism in mediating fibroblast proliferation in progression of fibrotic lung disease [20].

**Figure 3:**
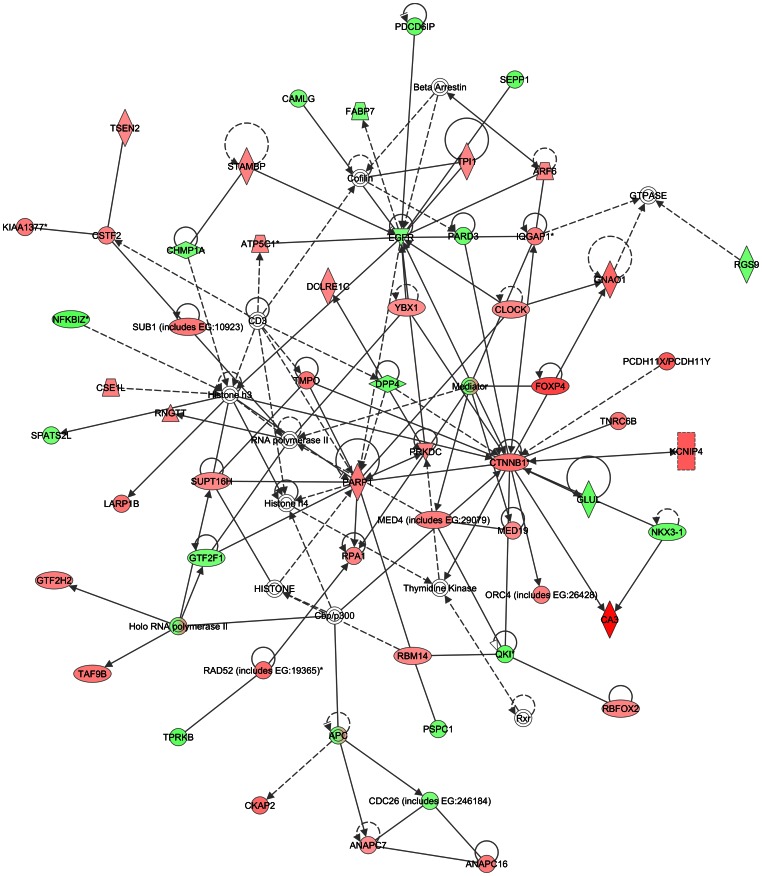
Network analysis of growing tumors compared to non-growing tumors – VHL hub. Symbols in the figure represent the following: **Rectangle:** Nuclear receptor. **Oval:** Transcription regulator **Triangle:** Phosphatase. **Horizontal diamond:** Peptidase **Vertical diamond**: Enzyme. **Trapezium:** Transporter **Square with solid boundary:** Cytokine. **Square with dashed boundary:** Growth factor **Circle:** Other. **Solid line:** Direct interaction **Dashed line:** Indirect interaction. **Arrow:** Acts on **Horizontal “T”:** Inhibits.

Two other networks identified as significant in our data have been noted to be important for breast cancer. The first is centered on up-regulation of *PARP1*, the other on the down-regulation of *EGFR* (Figure 3). *PARP1* has roles in DNA damage response, reinitiation of stalled replication forks and is up-regulated in many cancers including breast cancer [21]. Connecting with the *PARP1* hub in leiomyoma from older black women were other genes involved in transcription or DNA repair including *PRKDC*, catenin (cadherin-associated protein), beta 1subunits, *MED4*, *MED19* and *MEDIATOR* complex. Recently, *MED12* mutation was reported in a high percentage of leiomyoma [1] and has direct interactions with beta catenin, as does *MED19* [22]. Interestingly, the specificity of this complex in mediating gene transcription is attributed in part to the relative amount or activity of each subunit and it may be that subunits overlap and substitute functions. While these networked transcription and DNA repair genes are up-regulated in leiomyoma from older black women, EGFR networked genes were generally down-regulated, different from its growth promoting networks in breast cancer. It is also of interest that *EGFR* was only identified in the leiomyoma to leiomyoma comparison, while *ERBB2* was identified only in leiomyoma compared to myometrium. The differentiation of these genes suggests a refinement of their roles in tumorigenesis.

## Discussion

The study of uterine leiomyoma presents a unique opportunity to expose possible fundamental characteristics about the molecular and physical heterogeneity of hormone-dependent tumor growth with relevance to hormone-dependent malignant cancers. Previous gene expression studies of uterine leiomyomata have compared tumors to matched myometrium often with sub-classifications of leiomyoma based on size, location, or menstrual cycle phase to combine groups assumed to have similar characteristics [4],[23],[24],[25],[26],[27]. Certainly such comparisons have revealed important pathways that distinguish leiomyoma from myometrium, and our microarray analysis comparing leiomyoma to smooth muscle corroborate these findings, despite using different software analysis tools [6],[28],[23] and despite a limited number of myometrial samples. Indeed, compared to results tabulated by Arslan et al, our data set was most consistent across all studies, matching 24 of 38 genes up-regulated and 37 of 43 down-regulated (Table S5 and S6). Our gene expression studies were also validated by the RT-PCR results of genes selected a priori showing consistent up-regulation of many hormone receptor-related genes and the down-regulation of genes involved in prostaglandin synthesis, a finding also reported by Arslan, et al, 2005 [6].

The contributions of our analysis were to identify genes and signaling pathways potentially involved in tumor growth based on statistical modeling results of a detailed MRI study of tumor volume changes over time. Indeed, the signaling pathways identified in the comparison between tumors from older black and older white women are well-recognized in tumorigenesis, particularly those involved in transcriptional regulation and hypoxia, but had not been defined in any other leiomyoma gene expression comparisons. The fact that the comparison is between two ethnic groups may imply a genetic contribution to the data sets, yet, does not subtract from the basic premise that specific genes identified in these comparisons likely play a role in tumor growth.

The relevance of genes and pathways identified by this approach is both supported by and substantiates experimental data. For example, *MED12* down-regulation was identified in the comparison of tumors and normal tissue, while the Mediator complex and subunits *MED4* and *MED19* were specifically identified in the IPA analysis of the older black/older white comparisons of leiomyoma. These findings suggest there may be specificity to global transcriptional regulation and epigenetic controls relating to growth. In contrast, *IGF1R* and related signaling pathways, classically defined as growth promoting, were not differentially expressed between the two leiomyoma groups, but were up-regulated in leiomyoma compared to myometrium as in other studies of leiomyoma [6]. Such findings implicate IGF-related pathways not as growth promoting factors in leiomyoma, but as important survival signals. Indeed, this conclusion is consistent with experimental data which shows that in cultured leiomyoma cells IGF1 pathways function, in concert with estrogen, as survival signals with little or no effect on cell proliferation [29].

Canonical pathways identified in the comparisons of the two leiomyoma groups all related to processes of cell survival consistent with the idea that leiomyoma growth is promoted by an overall inhibition of apoptosis pathways. Contribution of cell survival pathways, as opposed to cell proliferation, is consistent with the generally low mitotic index of leiomyoma. While there is no question that much of the mass of a leiomyoma is due to the collagenous extracellular matrix, we propose that the increase in cellular mass of leiomyoma in older black women most likely to be growing is due to an accumulation of cells that are resistant to tissue and cellular based death signals rather than due to an accumulating mass of actively proliferating cells. Alternatively, a fundamental molecular trigger in regressing leiomyoma may be induction of apoptosis and death signals, rather than due to inhibition of cell proliferation.

The severe hypoxic environment of leiomyoma in situ has been convincingly demonstrated by Mayer et al, 2008 [30], although the molecular constituents allowing cell survival were not fully established. In our study, the most highly expressed gene in leiomyoma likely to be growing and those more likely to be non-growing (leiomyoma from older blacks vs. from older whites) was a muscle specific cytoplasmic carbonic anhydrase (*CAIII*), an enzyme which serves to buffer the cellular acid-base balance (pH regulation) (reviewed in Breton et al., 2001 [13]). *CAIII* is up-regulated in response to increased lactate production in hypoxic conditions with such consistency that it is often used as a surrogate marker for hypoxia-induced pathways. A unique trait of smooth muscle metabolism is a continual production of lactate even under normoxia [31]. Thus, the programming of smooth muscle cell metabolism provides a growth advantage for these cells to proliferate in an acidic and hypoxic environment. Recently, Norian et al (2011) [32] reported on the reorganization of the actin cytoskeleton mediated by RhoA in leiomyoma cells, confirming the link between RhoA activation in leiomyoma, while Turcotte et al., 2004 [15], demonstrated that hypoxic up-regulation of *VHL* is a Rho-A mediated event. Taken together, these data suggest a scenario in which smooth muscle cells within the leiomyoma have the innate capacity to survive within an acidic and hypoxic environment and within this microenvironment are resist apoptotic signals because of aberrant cytoskeletal alterations.

If growth of leiomyoma is mediated through aberrant cytoskeletal alterations under conditions of low oxygen or hypoxia, then there are numerous events throughout the reproductive cycle which would initiate and promote the formation of uterine leiomyoma. Both experimental and epidemiological data support the idea that pregnancy but, particularly, parturition and post-partum remodeling, is growth suppressive and growth repressive for uterine leiomyoma [33],[34]. Under physiological conditions uterine smooth muscle proliferation and hypertrophy occur during pregnancy, at which time cells are also stimulated by stretch signals imparted by the developing fetus. This biomechanical loading is delivered to the cytoskeleton through integrin-containing cell-matrix adhesions [35], [36] and, coupled with growth signals, permits organized proliferation and deposition of extracellular matrix for uterine volumetric increase in size and strength to accommodate the growing fetus. Parturition is characterized as a remarkably ischemic event followed by extensive uterine remodeling and involution. Thus, conditions that maintain an organized signaling matrix followed by programmed contraction and ischemia promote normal smooth muscle function. However, aberrant oxygen tension or misappropriated biomechanical loading of smooth muscle cells at any time, including during pregnancy or parturition, could set the stage for the development of a leiomyoma. Indeed, hypoxic states during the pre- and periparturient periods are associated with uterine acidification, abnormal calcium signaling and loss of contractile apparatus in smooth muscle cells – also a phenotype of leiomyoma cells.

Recently, a population of myometrial stem cells have been identified within the nongravid uterus proposed to contribute to the growth of the myometrium during pregnancy [37]. These cells are reported to underexpress or lack myometrial cell markers, proliferate and eventually differentiate into mature myometrial cells *in vitro* only under low oxygen concentration. Hypoxia appears to influence cell-fate commitment and proliferative capacity in many stem cells. Intriguingly, a role for Mediator complex has been indentified in epigenetic regulation of embryonic stem cell renewal, pluripotency and differentiation. Whether leiomyomata are derived from stem cells or from existing myometrial cells, our analyses support a model for growth of uterine leiomyoma that is an adaptive self-promoting response to hypoxia within uterine tissue.

Our analyses of uterine leiomyomata have broader implications as well, in that identifying potential molecular underpinnings of growth control in a benign diploid hormone dependent tumor of the uterus helps provide a basis for understanding the growth controls in more complex endocrine-sensitive malignant cancers of the female reproductive system. We show numerous genes and signaling pathways implicated in epithelial mesenchymal transition, yet leiomyoma are a benign fibrogenic mesenchymal tumor. Certainly the study of benign diseases has led to unexpected discoveries and insights relevant to malignant cancers, for example, in studying hamartomas associated with Cowden's and Tuberulo Sclerosis syndromes and the identification of *PTEN* and *TSC2* genes, respectively. We submit that the study of uterine leiomyomata provides equal opportunities for accelerating our understanding of the pathogenesis of malignant endocrine-sensitive cancers in women.

## Methods

### Sample collection

With respect to the analysis herein, about one-third of the FGS participants elected to have either a hysterectomy or myomectomy and had consented to donate tumor tissue and normal myometrium at the time of surgery. Details regarding the sample sizes are provided in Table 1. Study participants underwent an MRI prior to surgery, and radiologic descriptions were recorded for each tumor, including numerical identification, anatomical position, dimensions, homogeneity of muscle tissue and estimated percentage of calcification, hemorrhage, necrosis, and cystic change. This research was approved by the institutional review boards of the National Institute of Environmental Health Sciences and the University of North Carolina. The study participants provided signed informed consent. If hysterectomy was performed, the uterus was evaluated by a pathologist and leiomyoma samples were identified and collected with reference to their MRI images. In the case of myomectomy, the surgeon identified the tumors in situ with respect to the MRI, and specific leiomyoma removed were labeled as per surgeon's description. For tumors 5 cm or larger, 1-cm^3^ samples were collected from the anterior pole, posterior pole, right side, left side, and center and quick frozen in liquid nitrogen. For tumors less than 5 cm, samples were collected from the same five locations to the extent possible without compromising the sample size. Samples of normal uterine tissue were collected from hysterectomy patients in areas adjacent to leiomyoma and from the endometrial, intramural, and serosal layers at a location distant from the leiomyoma within the uterus.

### Microarray gene expression analysis

The gene expression profiles of leiomyoma and normal myometrium were assessed by using the Human Genome U133 plus 2.0 microarray technology from Affymetrix Gene Chip expression arrays (54,675 array features covering >28,000 UniGene clusters). Five micrograms of total RNA from each sample were labeled using the bioarray high-yield transcript kit according to manufacturer's conditions (ENZO, Farmingdale, NY). Labeled RNAs were hybridized and washed according to manufacturer's recommendations (Affymetrix, Inc., Santa Clara, CA). Initial gene expression data analysis was done using Microarray Suite 5.0 software (Affymetrix 2001). All arrays were normalized to a trimmed mean transcript signal level of 500 counts (absent and present call procedure).

### Statistical analysis of gene expression data

The log base 2 expression data (perfect match only) were normalized using the systematic variation normalization method [38]. The normal samples were used as a reference to adjust the other arrays. The data were summarized by summing the data for the probe pairs. Principal component analysis (PCA) and hierarchical clustering were performed using the Partek Genomics Suite Software (version 6.5). For hierarchical clustering, the data were standardized to mean  = 0, variance  = 1 and grouped using Euclidean distance as the dissimilarity measure and average linkage for merging. Since the data are not necessarily normally distributed with equal variances, and since the sample sizes are unequal among the comparison groups, we performed all comparisons using standard residual bootstrap methodology [39] implemented in ORIOGEN v.3.0 which is based on [40], [41] using 100000 bootstrap samples with a SAM correction of 0.10. Specifically, we compared samples from normal and tumor tissues, tumor tissues from older black and older whites, and tumor tissues from older and younger black women. A false discovery rate of 0.05 was used in identifying differentially expressed genes.

### Down-stream analyses of differentially expressed genes

Genes that were found significantly different in a pairwise comparison were further analyzed for canonical pathways, networks, transcription factors and biological functions using the Ingenuity Pathway Analysis (IPA) software (*Ingenuity Systems, Redwood City, CA, USA*). The IPA software is based on computational algorithms of the connectivity from information obtained within the IPA database. IPA analysis accounts for the type of chip used with a score assigned to rank networks according to their relevance to the gene list provided. Canonical pathways were determined by analyzing a ratio of the number of genes that map to the pathway, divided by the total number of genes in the pathway that are represented by the chip probes, and the *p* value calculated by Fisher's exact test to determine the probability that the association was due to chance alone.

### Quantitative Real Time PCR (QRT-PCR) Assays

Genes analyzed for qRT-PCR assays were selected a priori based on our previous studies testing the hypothesis that uterine smooth muscle tumor cells mimic a differentiated myometrial cell of pregnancy with a hypersensitivity to sex steroid hormones and the inability to respond to apoptotic or dedifferentiation signals mediated by prostaglandins [7], [42]. The analysis was performed from the same samples submitted for microarray analysis. Frozen tissue samples were processed according to standard protocols using Qiagen RNeasy Midi Columns and Fibrous Tissue Midi protocol (Qiagen Inc. Valencia, CA), on-column DNase treatment using the RNase-free DNase kit (Qiagen Inc. Valencia, CA). The RNA quality was assessed by Agilent software analysis (Agilent Technologies Santa Clara, CA). RNA was reversed transcribed according to manufacturer's protocols using the High-Capacity cDNA Archive kit in 2X RT Master-Mix containing Taqman RT buffer, random primers, dNTP mixture, and Multiscribe RT enzyme in 96 well reaction plate (all from Applied Biosystems, Foster City, CA). Samples were done in duplicate, as well as a no amplification control (NAC) (Table S7). Consensus sequence primer probe sets and four custom designed probe sets were ordered from Assays By Design^TM^ File Builder 2.0 (Applied Biosystems, Foster City, CA) (Table S8). Software version 2.1 (Applied Biosystems, Foster City, CA) and Microsoft Excel software was used for analysis of the resulting data from the relative quantitation assay. Manual threshold values were used and expression of each gene was normalized to the geometric mean of Actin-β (ACTB), hypoxanthine phosphoribosyltransferase 1 (HPRT1), and Eukaryotic 18S rRNA (18S). Fold change was calculated as described by K. Livak [43]. All the Primer Probe sets were tested for reaching efficiency using human uterus total RNA from Ambion (Applied Biosystems, Foster City, CA).

## Supporting Information

Figure S1 **Principal component analysis plot of the gene expression data of leiomyoma samples (blue balls) and normal myometrium samples (red balls).** Left panel is based on all probes on the chip and the right panel is based on differentially expressed probes.
(TIFF)

Figure S2 **Principal component analysis plot of the gene expression data of leiomyoma samples from aged black women (red balls) and aged white women (blue balls).** Left panel is based on all probes on the chip and the right panel is based on differentially expressed probes.
(TIFF)

Figure S3 **Heat map based on probes determined to be significantly different between the aged black and aged white.** The data (probe-wise) were standardized to a mean of 0, standard deviation of 1 and then then clustered using the cosine correlation as a dissimilarity measure and average linkage for hierarchical grouping.
(TIFF)

Table S1 **Genes found significantly up-regulated in uterine leiomyoma compared to myometrium from FGS.**
(DOCX)

Table S2 **Genes found significantly down-regulated in leiomyoma vs. myometrium in FGS.**
(DOCX)

Table S3 **List of all differentially expressed genes between leiomyoma and myometrium in FGS.**
(XLS)

Table S4 **Quantitative gene expression of** ***a priori*** **selected genes in leiomyoma compared to expression from microarray analysis.**
(DOCX)

Table S5 **Comparison of up-regulated genes in leiomyoma compared to myometrium from various studies.**
(DOCX)

Table S6 **Comparison of down-regulated genes in leiomyoma compared to myometrium from various studies.**
(DOCX)

Table S7 **Taqman Primer Probe Sets.**
(DOCX)

Table S8 **Custom-designed primer probe sets.**
(DOCX)
