# Supplementary figures and images for: Gene Expression in Uterine Leiomyoma from Tumors Likely to Be Growing (from Black Women over 35) and Tumors Likely to Be Non-Growing (from White Women over 35)
Source: PLoS One. 2013 Jun 13;8(6):e63909. doi: 10.1371/journal.pone.0063909 (PMC3681799; doi:10.1371/journal.pone.0063909)

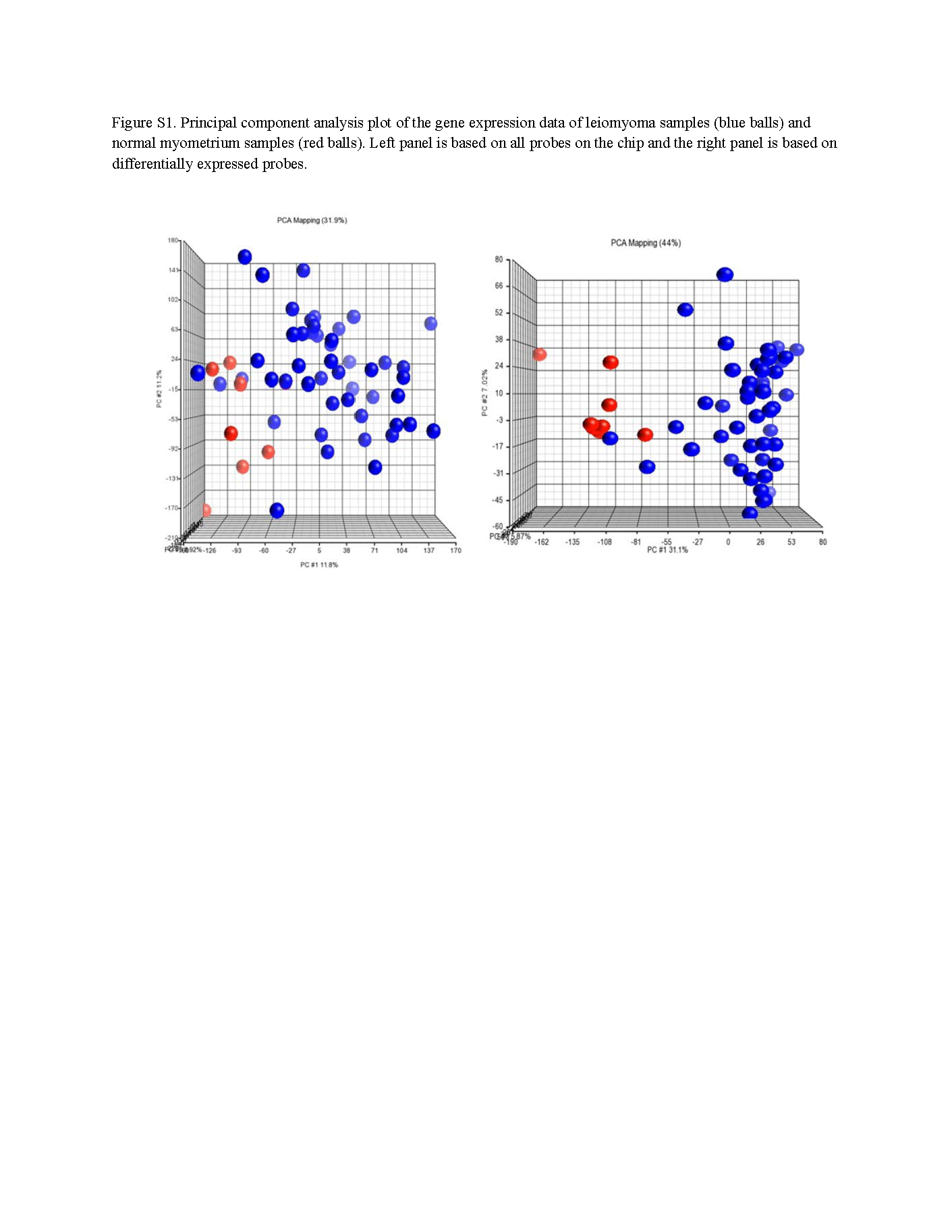

Supplement: Figure S1 — Principal component analysis plot of the gene expression data of leiomyoma samples (blue balls) and normal myometrium samples (red balls). Left panel is based on all probes on the chip and the right panel is based on differentially expressed probes. (TIFF) [file pone.0063909.s001.tiff]

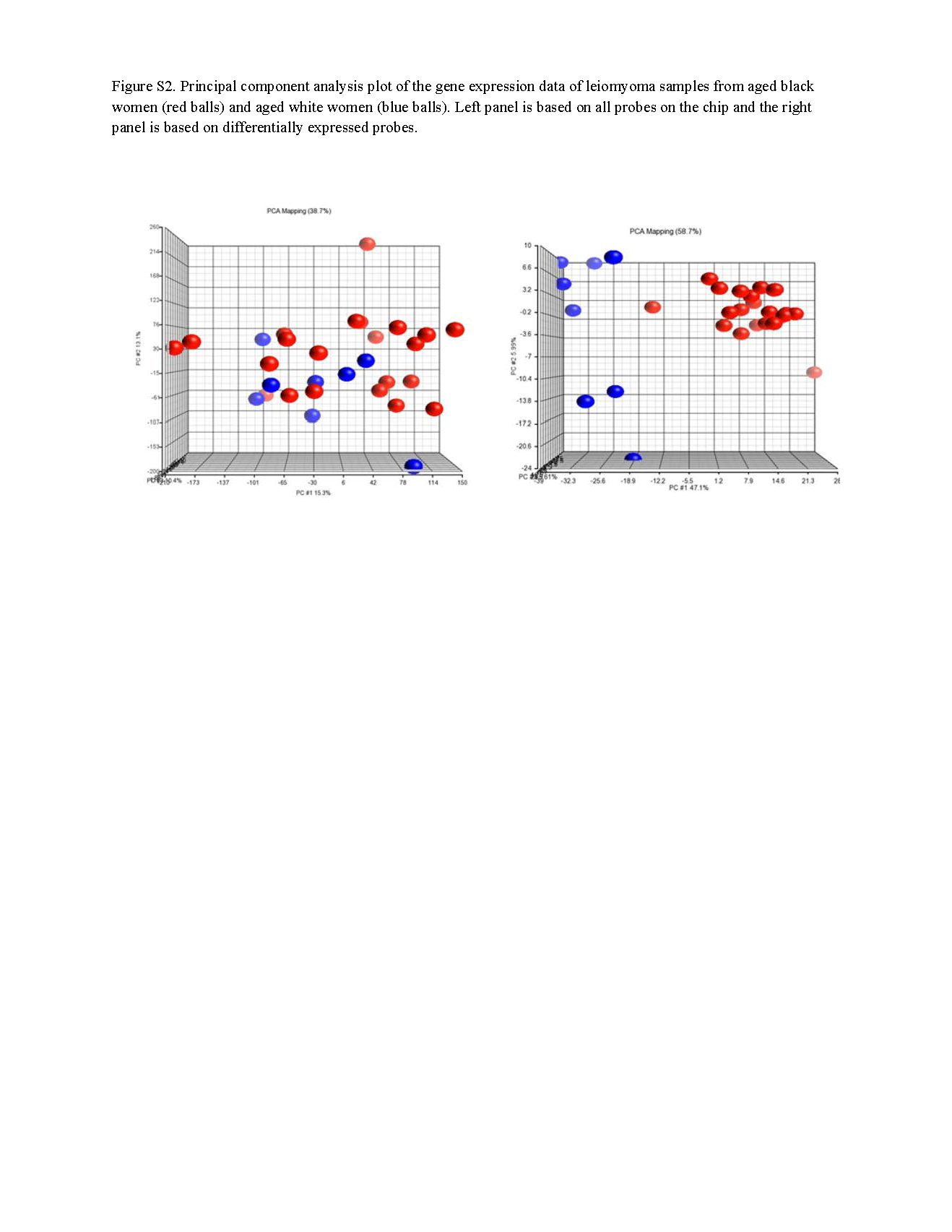

Supplement: Figure S2 — Principal component analysis plot of the gene expression data of leiomyoma samples from aged black women (red balls) and aged white women (blue balls). Left panel is based on all probes on the chip and the right panel is based on differentially expressed probes. (TIFF) [file pone.0063909.s002.tiff]

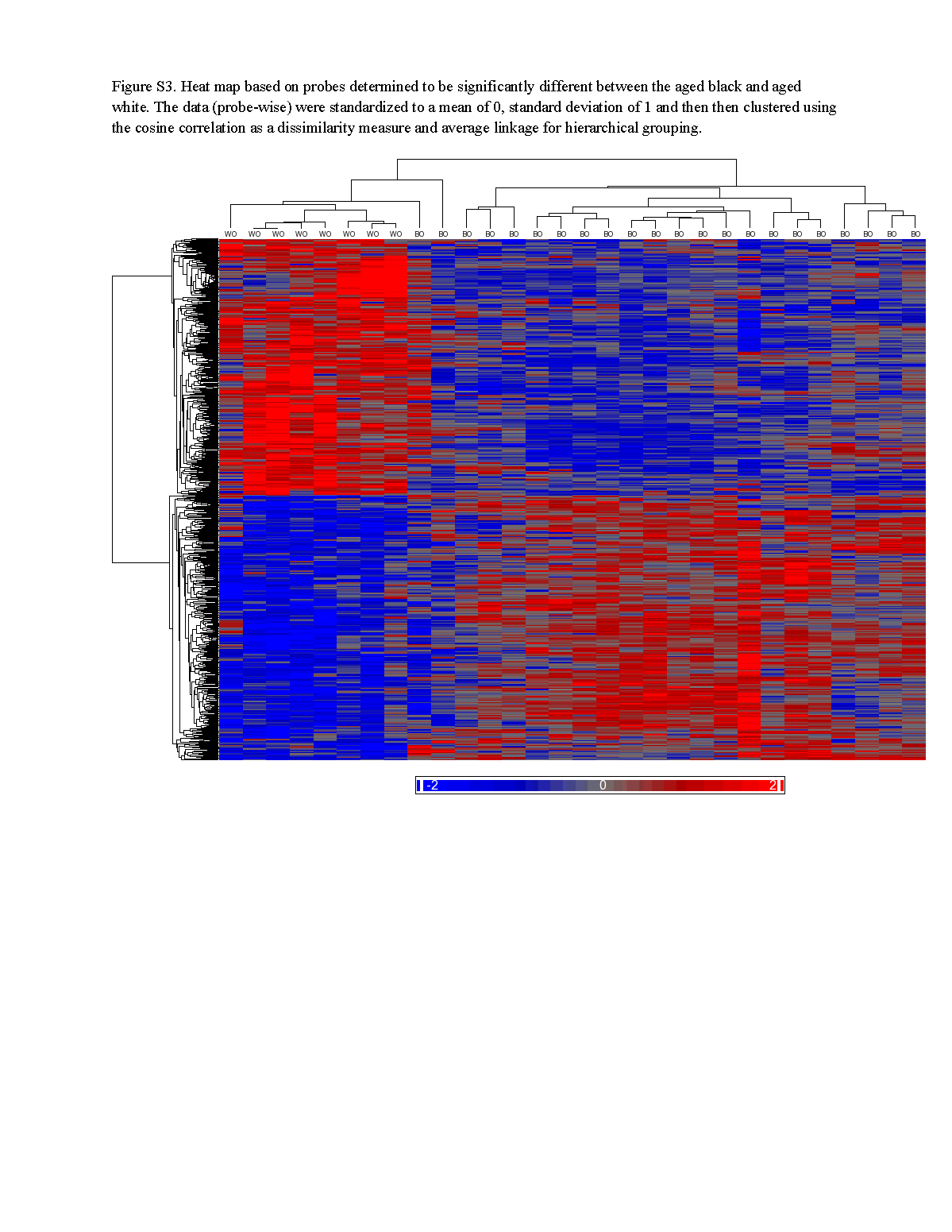

Supplement: Figure S3 — Heat map based on probes determined to be significantly different between the aged black and aged white. The data (probe-wise) were standardized to a mean of 0, standard deviation of 1 and then then clustered using the cosine correlation as a dissimilarity measure and average linkage for hierarchical grouping. (TIFF) [file pone.0063909.s003.tiff]
