# Supplementary material for: Gene Expression in Uterine Leiomyoma from Tumors Likely to Be Growing (from Black Women over 35) and Tumors Likely to Be Non-Growing (from White Women over 35)
Source: PLoS One. 2013 Jun 13;8(6):e63909. doi: 10.1371/journal.pone.0063909 (PMC3681799; doi:10.1371/journal.pone.0063909)
Supplement: Table S2 — Genes found significantly down-regulated in leiomyoma vs. myometrium in FGS. (DOCX) [file pone.0063909.s005.docx]

Table S2. Genes found significantly down-regulated in leiomyoma vs. myometrium in FGS
