# Supplementary material for: Gene Expression in Uterine Leiomyoma from Tumors Likely to Be Growing (from Black Women over 35) and Tumors Likely to Be Non-Growing (from White Women over 35)
Source: PLoS One. 2013 Jun 13;8(6):e63909. doi: 10.1371/journal.pone.0063909 (PMC3681799; doi:10.1371/journal.pone.0063909)
Supplement: Table S4 — Quantitative gene expression of a priori selected genes in leiomyoma compared to expression from microarray analysis. (DOCX) [file pone.0063909.s007.docx]

Table S4. Quantitative gene expression of *a priori* selected genes in leiomyoma compared to expression from microarray analysis

| **Gene Symbol** | **GENE NAME** | **RT-PCR RESULTS** | **Array Fold Change** |
| --- | --- | --- | --- |
| PRLR | Prolactin Receptor | 1.91 | 2.82 |
| LEPR | Leptin Receptor | -2.14 | -3.08 |
| PTGS1 | Prostaglandin-endoperoxide Synthase 1 (cyclooxygenase I) | -1.55 | -1.25 |
| COL2A1 | Collagen, type II, alpha 1 | 65.53 | 3.25 |
| PTGS2 | Prostaglandin-endoperoxide Synthase 2 (cyclooxygenase 2) | -4.88 | -5.41 |
| COL4A6 | Collagen, type IV, alpha 6 | 2.46 | 1.78 |
| COL4A3 | Collagen, type IV, alpha 3 | -9.53 | -1.81, 1.17 |
| ESR1 | Estrogen Receptor 1 | 2.18 | 1.73 |
| COL4A5 | Collagen, type IV, alpha 5 | 2.54 | 1.56 |
| COL1A2 | Collagen, type I, alpha 2 | 2.14 | 1.83 |
| ESR2 | Estrogen Receptor 2 (ER beta) | 1.78 | -1.28,1.29 |
| COL4A2 | Collagen, type IV, alpha 2 | 3.16 | 2.43 |
| COL1A1 | Collagen, type I, alpha 1 | 3.01 | 2.58 |
| PR-A* | Progesterone Receptor A* | 1.52 | 2.10 |
| COL4A1 | Collagen, type 4, alpha 1 | 2.85 | 2.37 |

***Array only lists PGR (progesterone receptor)**
