# Supplementary material for: Gene Expression in Uterine Leiomyoma from Tumors Likely to Be Growing (from Black Women over 35) and Tumors Likely to Be Non-Growing (from White Women over 35)
Source: PLoS One. 2013 Jun 13;8(6):e63909. doi: 10.1371/journal.pone.0063909 (PMC3681799; doi:10.1371/journal.pone.0063909)
Supplement: Table S5 — Comparison of up-regulated genes in leiomyoma compared to myometrium from various studies. (DOCX) [file pone.0063909.s008.docx]

Table S5.Comparison of up-regulated genes in leiomyoma compared to myometrium from various studies

| Symbol | Function | Tsibris | Wang | Skubitz | Ahn | Catherino | Quade | Hoffman | Arslan | FGS |
| --- | --- | --- | --- | --- | --- | --- | --- | --- | --- | --- |
| ACTC | Cell motility | 5.2 |  | 3.2 |  |  |  |  |  | 2.37 |
| APOE | Lipid metabolism | 20.3 |  | 2.9 |  |  |  |  |  | 2.02 |
| CA12 | Enzyme | 4.8 |  |  | 3.1 |  |  |  |  | 4.92 |
| CD24 | Selectin ligand | 8.5 |  | 2.7 |  | 15 |  |  | 5.2 | 34.46 |
| CDKN1A | Kinase inhibitor | 8 |  |  | 3.2 |  | 3.2 |  |  | 1.16 |
| COL4A6 | ECM protein |  |  |  | 3.2 |  | 2.1 |  |  | 1.78 |
| CRABP2 | RA-binding protein | 5.1 | 2.6 | 3.6 |  | 11.6 |  | 3.1 |  | 2.62 |
| CRMP1 | Invasion suppressor | 2.6 |  |  | 3 |  | 2.6 |  |  | 3.69 |
| CSPG2 | ECM protein | 3.8 |  |  |  |  |  |  | 3.4 | no |
| DCX | Cell motility | 51.4 |  | 6.9 | 4.3 |  |  | 5.6 |  | 14.49 |
| DLK1 | Lipid metabolism | 70 |  | 4.5 |  |  |  |  |  | 4.22 |
| ESR1 | Cell growth |  | 1.5 |  |  |  | 1.1 | 2.1 |  | 1.74 |
| FZD2 | Wnt receptor | 8.6 |  |  |  | 1.8 |  |  |  | 2.37 |
| GAGEC1 | Transcription factor | 40.7 |  |  | 10.4 |  |  | 3 | 3.6 | no |
| GRIA2 | Angiogenesis | 38.8 | 4.9 | 3.6 |  |  | 7.3 | 7.5 |  | 73.93 |
| HTR2B | Signal transduction |  |  |  |  |  | 1.4 |  | 4.2 | 2.68 |
| IGF2 | Growth factor | 16.8 | 2.6 | 2.9 | 2.2 | 13.5 |  | 3 | 3.5 | 5.53 |
| IL17 | Inflammation | 30.0^b^ |  | 3.1 |  |  |  |  |  | 5.71 |
| KIF5C | Cell motility |  |  | 2.8 | 6.2 |  |  |  |  | 3.59 |
| MEST | Growth factor | 11.6 | 2.4 | 3.1 |  |  |  | 3.9 | 6.1 | 6.68 |
| MMP11 | IGFBP protease | 5.1 |  |  |  |  |  |  | 5.8 | 11.32 |
| PCP4 | Unknown | 8.2 |  |  |  |  | 3.1 |  |  | 8.35 |
| PEG3 | Signal transduction |  | 2.5 |  |  |  |  | 2.4 |  | 2.63 |
| RODH | RA synthesis | 3.3 |  | 3.1 |  |  |  | 1.9 |  | no |
| SFRP1 | Angiogenesis |  |  |  |  |  |  | 3.5 | 4.8 | 4.83 |
| TGFB3 | Signal transduction | 3.4 |  |  |  |  | 3.4 |  |  | 2.68 |
| TMSNB | Angiogenesis | 5.5 |  |  |  |  |  |  | 5.3 | no |
| TYMS | Nucleic acid enzyme | 9.1 | 3.3 |  |  |  | 6.6 | 2.1 |  | 5.5 |
