# Supplementary material for: Gene Expression in Uterine Leiomyoma from Tumors Likely to Be Growing (from Black Women over 35) and Tumors Likely to Be Non-Growing (from White Women over 35)
Source: PLoS One. 2013 Jun 13;8(6):e63909. doi: 10.1371/journal.pone.0063909 (PMC3681799; doi:10.1371/journal.pone.0063909)
Supplement: Table S6 — Comparison of down-regulated genes in leiomyoma compared to myometrium from various studies. (DOCX) [file pone.0063909.s009.docx]

Table S6.Comparison of down-regulated genes in leiomyoma compared to myometrium from various studies

| Symbol | Function | Tsibris | Wang | Skubitz | Ahn | Catherino | Quade | Hoffman | Arslan | FGS |
| --- | --- | --- | --- | --- | --- | --- | --- | --- | --- | --- |
| ABCA | Lipid metabolism | −5.7 |  | −4.2 |  | −2.0 |  |  |  | -14.03 |
| ABLIM1 | Cell integrity | −4.3 |  |  | −3.4 |  |  |  |  | -5.2 |
| ADH1 | RA synthesis | −40.0 | −16.6 | −5.8 | −18.6 | −9.2 | −10.0 |  | −9.4 | -13.23 |
| ALDH1 | RA synthesis | −8.0 | −3.8 |  | −12.4 |  |  | −3.5 |  | -9.98 |
| ANXA1 | Chemotaxis | −4.3 |  |  | −4.8 |  |  | −4.1 |  | -5.03 |
| APM2 | Lipid metab. |  |  | −3.6 | −3.3 |  |  |  | −6.6 | no |
| APOD | Lipid metab. | −3.4 |  |  | −7.8 |  | −1.02 |  |  | -3.16 |
| ATF3 | Transcrip. factor | −6.0 |  | −4.0 |  | −2.8 | −16.7 | −8.8 | −5.2 | -14.6 |
| C7 | Compl. factor | −3.9 |  |  | −4.8 |  |  |  |  | -2.83 |
| CAV2 | Signal transd. |  |  |  |  |  |  | −2.5 | −2.4 | -1.97 |
| CCL14 | Signal transd. |  | −11.0 |  |  |  | −3.1 |  |  | -5.72 |
| CCL21 | Chemotaxis | −11.6 | −8.5 |  | −3.9 |  |  |  |  | no |
| CFH | Compl. factor | −5.3 |  |  | −3.8 |  |  |  |  | -2.49 |
| CITED2 | Transcrip. factor |  |  |  |  |  |  | −3.2 | −3.4 | -4.23 |
| CPA3 | Mast cell enzyme | −8.2 |  |  |  |  |  |  | −3.5 | -5.02 |
| CTGF | IGFBP/angiogen. |  |  |  | −4.2 |  | −2.9 | −2.7 | −2.9 | -2.39 |
| CYR61 | IGFBP/angiogen. | −5.3 |  |  | −4.9 |  | −6.3 | −5.8 | −5.1 | -5.78 |
| DPT | TGF-β modulator | −18.7 | −4.0 |  | −11.0 | −4.3 |  |  | −7.4 | -6.19 |
| DUSP1 | Kinase inhibitor |  |  |  | −3.0 |  | −3.4 | −7.4 | −3.8 | -4.94 |
| EFEMP1 | Cell adhesion | −4.5 |  |  |  |  |  |  | −4.5 | -11.03 |
| EMP1 | Plasma memb. |  |  |  | −2.4 |  |  | −3.7 | −3.9 | -3.47 |
| FOS | Transcrip. factor | −8.2 |  | −4.3 |  |  |  | −9.5 | −5.7 | -10.97 |
| FY | Plasma memb. |  |  |  | −3.5 |  |  | −2.5 | −6.7 | no |
| GATA2 | Transcrip. factor |  | −2.2 |  | −3.9 |  |  |  |  | -6.03 |
| GBP2 | Nucleic acid binding | −5.5 |  |  | −1.4 |  |  |  |  | -1.93 |
| HBB | Oxygen transport | −4.4 |  |  |  |  |  |  | −5.8 | -5.2 |
| IGFBP6 | IGFBP | −5.3 |  | −3.4 |  |  |  | −4.5 | −4.5 | -5.34 |
| JUN | Transcrip. factor |  |  | −5.0 |  |  | −2.9 |  | −3.2 | -4 |
| JUNB | Transcrip. factor |  |  |  |  |  | −3.4 | −4.4 |  | -3.83 |
| KCNB1 | Cell metab. |  |  |  |  |  | −1.1 |  | −5.0 | no |
| KRT19 | Cell integrity | −8.1 |  | −3.6 |  |  |  |  |  | no |
| MAP3K5 | Apoptosis | −4.1 | −3.0 |  |  |  |  | −2.1 |  | -1.87 |
| MCL1 | Apoptosis |  |  |  |  |  |  | −3.0 | −2.3 | -1.34 |
| PTGDS | Pg synthesis | −3.5 | −2.1 |  |  |  | −2.1 | −2.0 |  | -3.23 |
| PTGER3 | Pg receptor | −4.2 | −2.8 |  |  | −3.9 | −1.2 |  |  | -4.14 |
| RNASE4 | Nucleic acid binding | −3.4 |  |  |  |  |  |  | −2.1 | -2.41 |
| S100A4 | Signal transd. |  |  |  |  |  |  | −2.4 | −3.2 | -1.77 |
| S100A13 | Signal transd. |  | −1.6 |  |  |  | −1.5 |  |  | -4.11 |
| TGFBR2 | Signal transd. | −2.0 |  |  |  |  | −1.3 | −1.8 |  | -2.32 |
| TIMP3 | ECM regulation |  |  |  |  |  | −1.3 | −2.1 | −2.9 | -1.88 |
| TM4SF1 | Cell integrity |  |  |  |  |  |  | −3.5 | −3.4 | -3.25 |
| TPSB2 | Mast cell enzyme | −24.3 |  |  |  |  |  |  | −4.9 | -8.79 |
| ZFP36 | Cell metab. |  |  |  |  |  | −2.6 |  | −2.8 | -4.411 |
| # probe sets on array |  | 12 000 | 6800 | 12000 | 17000 | 33000 | 7000 | 22000 | 22000 | 45000 |
