# Supplementary material for: Gene Expression in Uterine Leiomyoma from Tumors Likely to Be Growing (from Black Women over 35) and Tumors Likely to Be Non-Growing (from White Women over 35)
Source: PLoS One. 2013 Jun 13;8(6):e63909. doi: 10.1371/journal.pone.0063909 (PMC3681799; doi:10.1371/journal.pone.0063909)
Supplement: Table S7 — Taqman Primer Probe Sets. (DOCX) [file pone.0063909.s010.docx]

Table S7: Taqman Primer Probe Sets

| **Gene Symbol** | **Gene Name** | **Accession Number** |
| --- | --- | --- |
| PRLR | Prolactin Receptor | NM_000949.2 |
| LEPR | Leptin Receptor | NM_002303.3 |
| PTGS1 | Prostaglandin-endoperoxide Synthase 1 (prostaglandin G/H synthase and cyclooxygenase) | NM_080591.1,NM_000962.2 |
| COL2A1 | Collagen, type II, alpha 1 (primary osteoarthritis, spondyloepiphyseal dysplasia, congenital) | NM_033150.1,NM_001844.3 |
| 18S | Eukaryotic 18S rRNA | X03205.1 |
| ACTB | Actin, beta | NM_001101.2 |
| PTGS2 | Prostaglandin-endoperoxide Synthase 2 (prostaglandin G/H synthase and cyclooxygenase) | NM_000963.1 |
| COL4A6 | Collagen, type IV, alpha 6 | NM_001847.1,NM_033641.1 |
| COL4A3 | Collagen, type IV, alpha 3 (Goodpasture antigen) | NM_031362.1,NM_031364.1 |
| CCNA2 | Cyclin A2 | NM_001237.2 |
| ESR1 | Estrogen Receptor 1 | NM_000125.2 |
| COL4A5 | Collagen, type IV, alpha 5 (Alport syndrome) | NM_033380.1,NM_033381.1,NM_000495.3 |
| COL1A2 | Collagen, type I, alpha 2 | NM_000089.3 |
| ESR2 | Estrogen Receptor 2 (ER beta) | NM_001437.1 |
| COL4A2 | Collagen, type IV, alpha 2 | NM_001846.1 |
| HPRT1 | Hypoxanthine phosphoribosyltransferase 1 (Lesch-Nyhan syndrome) | NM_000194.1 |
| REN | Renin | NM_000537.2 |
