# Supplementary material for: Gene Expression in Uterine Leiomyoma from Tumors Likely to Be Growing (from Black Women over 35) and Tumors Likely to Be Non-Growing (from White Women over 35)
Source: PLoS One. 2013 Jun 13;8(6):e63909. doi: 10.1371/journal.pone.0063909 (PMC3681799; doi:10.1371/journal.pone.0063909)
Supplement: Table S8 — Custom-designed primer probe sets. (DOCX) [file pone.0063909.s011.docx]

| **Assay ID** | **Forward Primer Sequence** | **Reverse Primer Sequence** | **Reporter 1 Sequence** |
| --- | --- | --- | --- |
| COL1A1 | CCTTCCTGCGCCTGATGT | TGCAGTGGTAGGTGATGTTCTG | CCACCGAGGCCTCC |
| PR-A | AGCCATTGGGCGTTCCA | CAACTGTATGTCTTGACCTGGTGAA | AAAGCCAAGCCCTAAGCC |
| PR-B | CTGGAGGCAGCAGTTCTAGTC | CGCCAACAGAGTGTCCAAGA | CAGTCCGCTGTCCTTTT |
| COL4A1 | CATTCTGCATCCTGGCTTGAAAA | CCGAATGTGCTTACGTGTGAAAATA | CAGCTCTGTTGAATCAC |

Table S8. Custom-designed primer probe sets
